# Predicting the outcomes of hepatocellular carcinoma downstaging with the use of clinical and radiomics features

**DOI:** 10.1186/s12885-023-11386-0

**Published:** 2023-09-12

**Authors:** Si-Yuan Wang, Kai Sun, Shuo Jin, Kai-Yu Wang, Nan Jiang, Si-Qiao Shan, Qian Lu, Guo-Yue Lv, Jia-Hong Dong

**Affiliations:** 1 Hepatopancreatobiliary Center, Beijing Tsinghua Changgung Hospital, School of Clinical Medicine, Tsinghua University, Beijing, China; 2 Research Unit of Precision hepatobiliary Surgery Paradigm, Chinese Academy of Medical Sciences, Beijing, China; 3 Department of Biomedical Engineering, School of Medicine, Tsinghua University, Beijing, China; 4 Department of Hepatobiliary and Pancreatic Surgery, General Surgery Center, First Hospital of Jilin University, Changchun, Jilin China

**Keywords:** Hepatocellular carcinoma, Downstaging, Predicting model, Machine learning, Radiomics

## Abstract

**Background:**

Downstaging of hepatocellular carcinoma (HCC) makes it possible for patients beyond the criteria to have the chance of liver transplantation (LT) and improved outcomes. Thus, a procedure to predict the prognosis of the treatment is an urgent requisite. The present study aimed to construct a comprehensive framework with clinical information and radiomics features to accurately predict the prognosis of downstaging treatment.

**Methods:**

Specifically, three-dimensional (3D) tumor segmentation from contrast-enhanced computed tomography (CT) is employed to extract spatial information of the lesions. Then, the radiomics features within the segmented region are calculated. Combining radiomics features and clinical data prompts the development of feature selection to enhance the robustness and generalizability of the model. Finally, we adopt the support vector machine (SVM) algorithm to establish a classification model for predicting HCC downstaging outcomes.

**Results:**

Herein, a comparative study was conducted on three different models: a radiomics features-based model (R model), a clinical features-based model (C model), and a joint radiomics clinical features-based model (R-C model). The average accuracy of the three models was 0.712, 0.792, and 0.844, and the average area under the receiver-operating characteristic (AUROC) of the three models was 0.775, 0.804, and 0.877, respectively.

**Conclusions:**

The novel and practical R-C model accurately predicted the downstaging outcomes, which could be utilized to guide the HCC downstaging toward LT treatment.

**Supplementary Information:**

The online version contains supplementary material available at 10.1186/s12885-023-11386-0.

## Background

Liver transplantation (LT) is a potentially curative treatment in patients with hepatocellular carcinoma (HCC), and the 5-year overall survival (OS) after LT was > 70% [1–4]. However, two-thirds of HCC patients did not meet the criteria for diagnosis [5, 6]. For advanced HCC patients, the median survival was 6–7 months if untreated, and the 5-year survival was 18–32% post-LT [3, 6–9]. Downstaging was defined as reducing the tumor burden to meet the criteria for LT, making it possible for patients beyond the criteria to have the chance of LT [4, 10, 11]. Recent studies have shown that 5-year OS of HCC patients after successful downstaging to Milan criteria was 73.2–93.8%, which was similar to patients initially meeting the Milan criteria and better than that of no transplantation (31.2%) [4, 12, 13]. The definition of successful downstaging was controversial, however, recent studies commended that the OS of HCC patients after successful downstaging to the setting criteria was similar to patients initially meeting the criteria [14–16].

Predicting the outcomes of downstaging could provide individualized treatment, reduce unnecessary interventions, and provide healthcare to HCC patients. Due to the wide range of downstaging failure rates 23–89%, there is an urgent requirement for an objective and accurate downstaging prediction model [16–18]. Some studies speculated that downstaging-failed patients had more frequent MVI and worse tumor grades than successful patients [14]. In the study by Barakat et al., the noninfiltrative expanding tumor type was the sole predictor of successful downstaging; however, noninfiltrative evaluation was difficult, especially in small tumors [3]. Overall, no specific objective models are available to predict the downstaging response precisely. Radiomics can deduce more information than human eyes and achieve brilliant prognostic accuracy in various clinical tasks, such as the prediction of microvascular invasion [19, 20]. Machine learning (ML) was used successfully in many applications related to classification (diagnosis), such as lung cancer and response to treatment. To the best of our knowledge, no previous studies have predicted the downstaging outcomes using ML in HCC patients.

In the current study, we developed a model based on clinical data and radiomics features to predict the downstaging success in HCC undergoing local regional or systemic therapy, which was validated in an independent test cohort. The present study aimed to establish an HCC downstaging prediction model based on clinical data and radiomics features through ML.

## Materials and methods

### Patients

Patients diagnosed with HCC who received locoregional therapy or systemic therapy for downstaging were considered for this study from March 2015 to December 2021 in Beijing Tsinghua Chunggung Hospital (Beijing, China) and from January 2019 to December 2021 in The First Hospital of Jilin University (Jilin, China). The diagnosis of HCC was based on radiographic imaging (Liver Imaging Reporting and Data System, LI-RADS) or biopsy. The inclusion criteria were as follows: (1) patients with baseline enhanced computed tomography (CT) and follow-up imaging 4–12 weeks after the first downstaging and complete clinical data; (2) the tumor was beyond the up-to-seven criteria. The exclusion criteria were as follows: (1) patients with cholangiocarcinoma and mixed hepatocellular-cholangiocarcinoma (diagnosed by pathology); (2) patients with metastasis. The successful downstaging in the current study was to facilitate LT. Based on the modified Response Evaluation Criteria in Solid Tumors (mRECIST) assessment, the endpoint of successful downstaging is that the patients meet the up-to-seven criteria for LT.

The clinical features consisted of demographic, laboratory parameters, and radiologic features. The demographic characteristics included age, sex, body mass index (BMI), hepatic virus infection, cirrhosis, ascites, Child-Pugh class, and chronic disease. Routine baseline laboratory examinations included white blood cells (WBCs), platelet count (PLT), hemoglobin (Hb), serum alpha-fetoprotein (AFP) level, Child-Pugh class, serum alanine aminotransferase (ALT), aspartate aminotransferase (AST), serum total bilirubin (TB), serum albumin (ALB), serum gamma-glutamyl transferase (GGT), prothrombin time (PT), and serum creatinine (Scr). The radiologic features included tumor number and macrovascular invasion. Enhanced CT scans were acquired using CT scanners. The slice thickness was 0.625–5 mm.

**Fig. 1:**
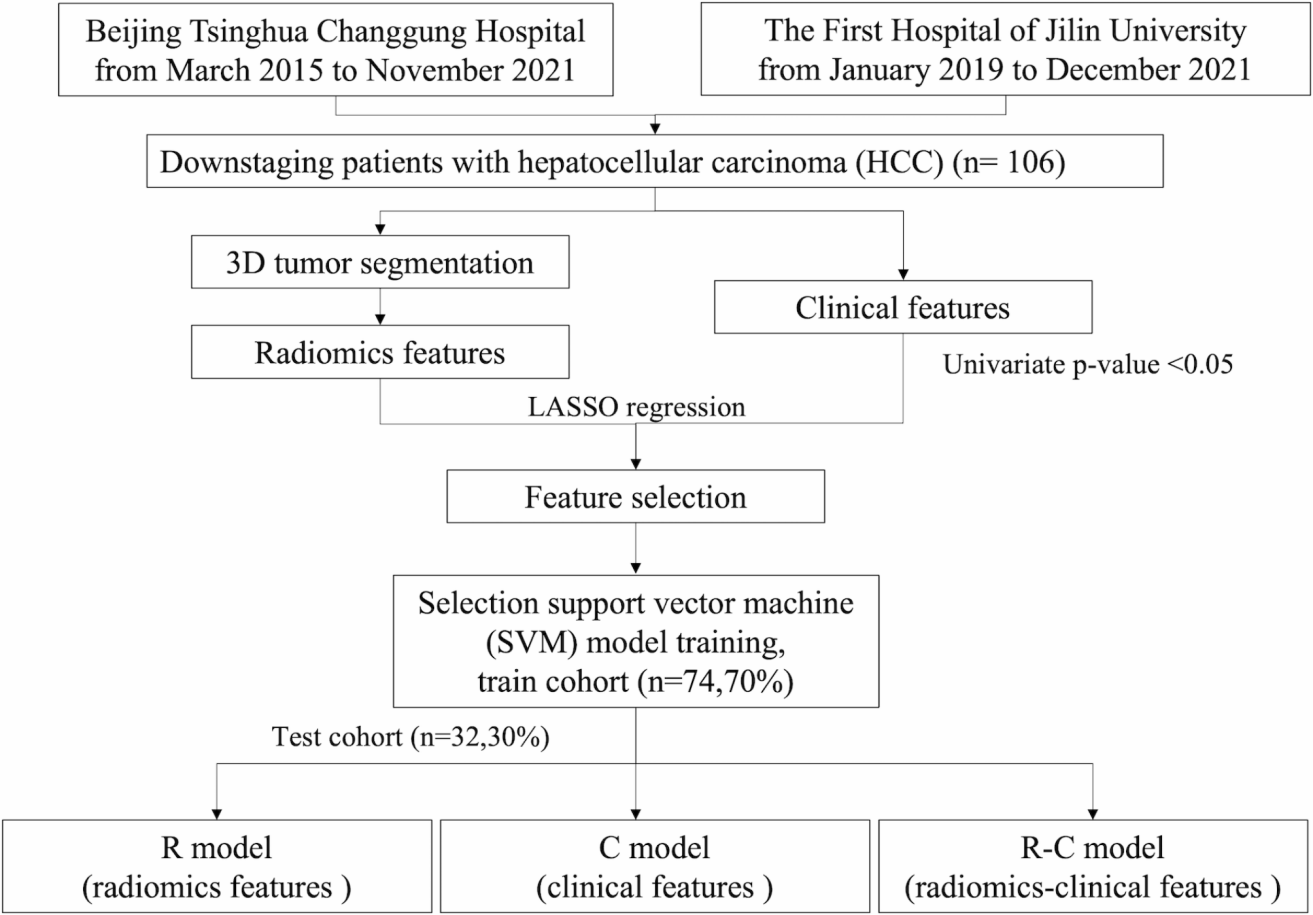
Patient recruitment workflow

The discovery cohort was randomly divided into two cohorts: the training set (n = 74, 70%) and the test set (n = 32, 30%) (see Fig. 1). The study was conducted according to the guidelines of the Declaration of Helsinki and approved by Beijing Tsinghua Changgung Hospital Ethics Committee (Approval No. 21269-4-04).

### Radiomics analysis

#### Tumor segmentation

**Fig. 2:**
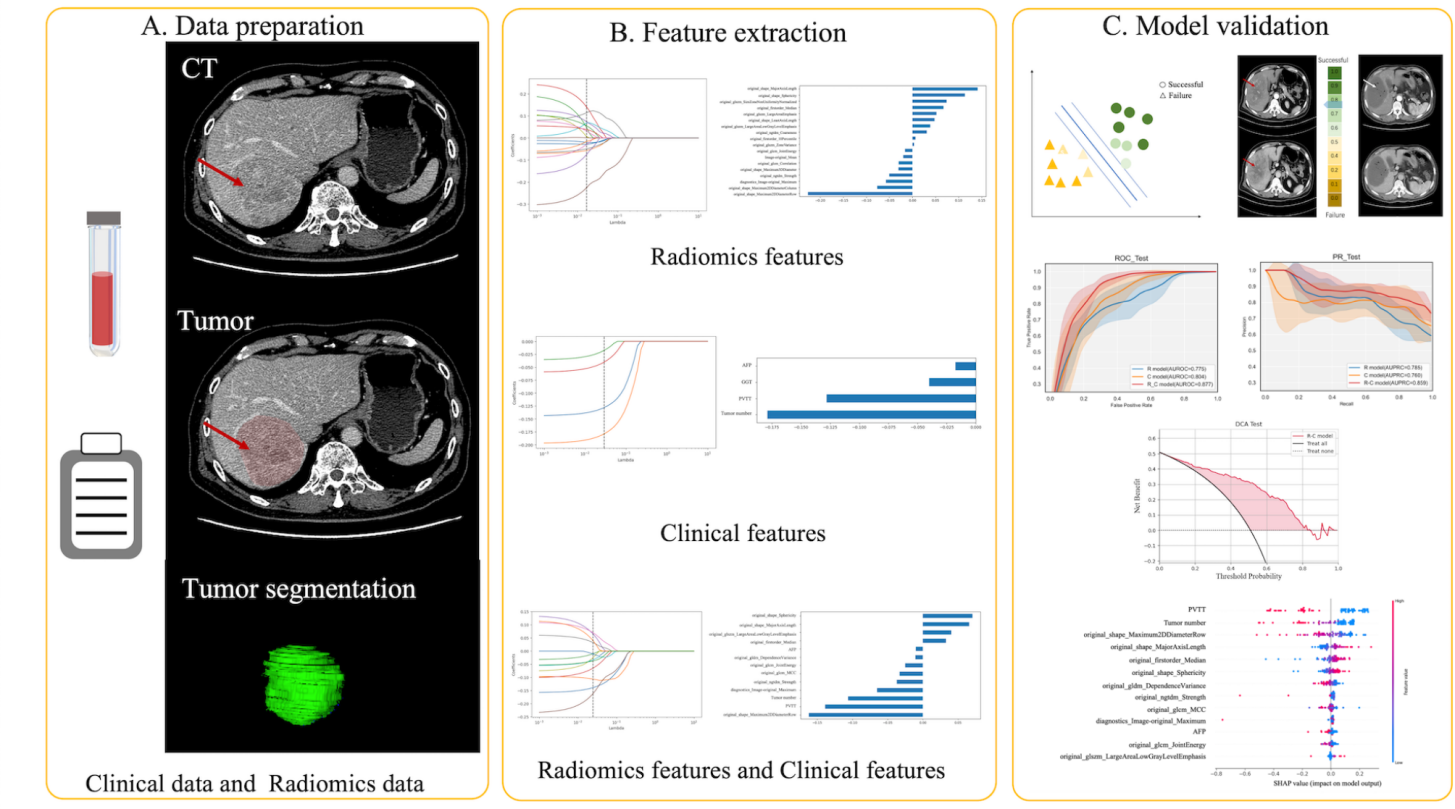
Model development overview (A) Data preparation (B) Feature extraction (C) Model validation

Tumor segmentation was performed by two experienced radiologists using ITK-SNAP (version 3.6.0) during the portal-venous phase of enhanced CT and reviewed by a senior radiologist, as shown in Fig. 2A.

#### Data pre-processing

The homogenous feature was calculated using default parameters. Since the CT images were heterogeneous, we performed image normalization to resample the CT volume to the same target spacing (1,1,1). Specifically, third-order spline interpolation was utilized for in-plane resampling, whereas nearest neighbor interpolation was performed for out-of-plane interpolation.

#### Features selection and model development

Radiomics features were extracted from each region of interest (ROI) using the pyradiomics package (https://www.cnpython.com/pypi/pyradiomics website, v3.0.1).

All features (including radiomics features and clinical information) were normalized to a standard scale based on z-scoring normalization.

$$X = \frac{{(x - \mu )}}{\sigma }$$

Where µ is the average value of all features, σ is the standard deviation of all features.

The least absolute shrinkage and selection operator (LASSO) regression model was used to select imaging features in high-dimensional data for the predictive model, as shown in Fig. 2B.

Next, we developed three models using only radiomics features (R), only clinical features (C), and both (R-C), respectively (see Fig. 2C). The classification prediction models were constructed based on a selection support vector machine (SVM) using the scikit-learn package.

Univariate analyses were performed to determine the clinical features. Clinical data that reached statistical significance in univariate analysis were included in the ML. In the model, we input missing values by the median of non-missing entries in the specific feature column. Finally, the candidate clinical variables and radiomics features were utilized to investigate the prediction model.

SHapley Additive exPlanations (SHAP) value was utilized to compute the distribution of features in the prediction model [21]. To enhance the interpretability of the model, we employed the SHAP value to express the importance of features in the established SVM classification model.

Normalization, LASSO, performance, and validation of the predictive model (confusion matrix and classification report) was assessed using scikit-learn packages (https://scikit-learn.org/stable/).

### Statistical analysis

Results were expressed as mean ± standard deviation and medians with interquartile range for continuous variables. Data were analyzed using the chi-square test, fisher’s exact test, t-test, or Mann–Whitney U test, as appropriate. True-positive (TP), true-negative (TN), false-negative (FN), and false-positive (FP) were considered variables for calculating performance metrics. The performance of the prediction model was quantified by accuracy, precision, and recall. Accuracy, precision, and recall were defined as follows:

$$Accuracy= \frac{TP+TN}{TP+FN+FP+TN}$$

$$Precision= \frac{TP}{TP+FP}$$

$$Recall= \frac{TP}{TP+FN}$$

In addition, the area under the receiver-operating characteristic curve (AUROC), the area under the precision-recall curve (AUPRC) and F1-score were utilized as evaluation indexes. F1 score was defined as:

$${F}_{1}= \frac{2TP}{FP+FN+2TP}$$

The clinical utility of the models was evaluated using decision curve analysis (DCA). The statistical comparison of AUROC was evaluated using Delong test. Statistical analyses were performed using SPSS v25.0 and R 4.3.2. Statistical significance was set at p < 0.05.

## Results

### Baseline clinical characteristics

A total of 106 HCC patients who underwent downstaging in Beijing Tsinghua Changgung Hospital from March 2015 to November 2021 and in The First Hospital of Jilin University from January 2019 to December 2021 were collected. The patients’ clinical features are listed in Table 1. This retrospective cohort comprised 95 males and 11 females with a mean age 56.36 ± 1.07 years. Also, 54 patients had successful downstaging, while the other 52 presented failed downstaging. The detailed downstaging treatments are listed in Table S1.

**Table 1:** Demographic and baseline data

Characteristics	No. of patients (n = 106)
Age (years) (± SD)	56.36(± 1.07)
Sex ratio (M: F)	95:11
BMI(kg/m^2^) (± SD)	24.70(± 0.32)
Hypertension	29(27.4%)
Diabetes mellitus	15(14.2%)
Viral hepatitis	88(83.0%)
Cirrhosis	65(61.3%)
Ascites	31(29.2%)
Child-Pugh class	
A	73(68.9%)
B	26(24.5%)
C	7(6.6%)
WBC(10^9^/L) (IQR)	5.27(3.93,6.63)
Hb(g/L) (IQR)	138.5(123.8,149.3)
PLT(10^9^/L) (IQR)	149.0(97.0,220.3)
Scr (µmol/L) n = 104(IQR)	63.30(56.38,73.53)
PT(s) (IQR)	12.75(11.90,14.10)
ALT(U/L) (IQR)	39.85(24.73,72.90)
AST(U/L) (IQR)	44.55(32.85,65.23)
TB(µmol/L) (IQR)	19.06(14.83,31.56)
GGT(U/L) (IQR)	111.20(56.00,199.20)
ALB(g/L) (± SD)	37.30(± 0.52)
AFP (ng/ml) n = 105	
<800	62(59.0%)
≥800	43(41.0%)
PVTT	
Yes	37(34.9%)
No	69(65.1%)
Type of treatment	
Locoregional therapy	88(83.0%)
Locoregional therapy and systemic therapy	18(17.0%)
Number of tumors	
1–2	63(59.4%)
3–5	20(18.9%)
>5	23(21.7%)
Outcomes of downstaging	
Successful	54(50.9%)
Failed	52(49.1%)

M, male; F, female; BMI, body mass index; WBCs, white blood cells; PLT, platelet count; Hb, hemoglobin; AFP, α-fetoprotein; ALT, alanine aminotransferase; AST, aspartate aminotransferase; TB, serum total bilirubin; ALB, albumin, GGT, gamma-glutamyl transferase; PT, prothrombin time; Scr, serum creatinine; PVTT portal vein tumor thrombus

The two groups were similar in their distribution of age, sex, BMI, hepatic virus infection, cirrhosis, ascites, chronic disease (hypertension and diabetes mellitus), and type of treatment (Table 2). Among all factors, the following clinical features were related to the downstaging outcomes in univariate analyses: Child-Pugh class, AST, GGT, AFP, Portal Vein Tumor Thrombus (PVTT), and the number of tumors (p < 0.05).

**Table 2:** Univariate analysis for different outcomes of downstaging

Variables	Successful(n = 54)	Failed(n = 52)	*P* value
Age (years) (± SD)	54.98(± 1.58)	57.79(± 1.43)	0.192
Sex ratio (M: F)	48:6	47:5	0.801
BMI(kg/m^2^) (± SD)	24.78(± 0.47)	24.60(± 0.45)	0.783
Hypertension			0.736
Negative	40(74.1%)	37(71.2%)	
Positive	14(25.9%)	15(28.8%)	
Diabetes mellitus			0.360
Negative	48(88.9%)	43(82.7%)	
Positive	6(11.1%)	9(17.3%)	
Viral hepatitis			0.667
Negative	10(18.5%)	8(15.4%)	
Positive	44(81.5%)	44(84.6%)	
Cirrhosis			0.214
Negative	24(44.4%)	17(32.7%)	
Positive	30(55.6%)	35(67.3%)	
Ascites			0.233
Negative	41(75.9%)	34(65.4%)	
Positive	13(24.1%)	18(34.6%)	
Child-Pugh class			0.021
A	40 (74.1%)	33(63.5%)	
B	8(14.8%)	18(34.6%)	
C	6(11.1%)	1(1.9%)	
WBC(10^9^/L) (IQR)	5.06(3.72,6.09)	5.79(4.36,7.24)	0.051
Hb(g/L) (IQR)	135.0(124.0,150.3)	140.0(121.3,148.8)	0.716
PLT(10^9^/L) (IQR)	147.0(97.0,215.5)	154.5(95.0,221.8)	0.945
Cr(µmol/L) n = 104(IQR)	67.40(56.65,76.50)	62.10(56.30,66.90)	0.088
PT(s) (IQR)	12.6(11.8,14.0)	12.9(12.3,14.1)	0.368
ALT(U/L) (IQR)	37.55(23.48,75.40)	40.95(25.03,67.23)	0.745
AST(U/L) (IQR)	36.50(26.93,59.15)	53.20(37.13,73.50)	0.001
TB(µmol/L) (IQR)	17.25(13.38,25.31)	21.87(16.15,32.13)	0.063
GGT(U/L) (IQR)	73.05 (47.23,147.10)	157.55(95.98,299.00)	< 0.001
ALB(g/L) (IQR)	39.15(33.03,42.40)	36.25(32.78,39.68)	0.067
AFP (ng/ml) n = 105			0.015
<800	38(70.4%)	24(47.1%)	
>=800	16(29.6%)	27(52.9%)	
PVTT			< 0.001
Negative	46(85.2%)	23(44.2%)	
Positive	8(14.8%)	29(55.8%)	
Number of tumors			< 0.001
1–2	42(77.8%)	21(40.4%)	
3–5	12(22.2%)	8(15.1%)	
>5	0(0.0%)	23(44.2%)	
Type of treatment			0.101
Locoregional therapy	48(86.5%)	40(76.9%)	
Locoregional therapy and systemic therapy	6(13.5%)	12(23.1%)	

M, male; F, female; BMI, body mass index; WBCs, white blood cells; PLT, platelet count; Hb, hemoglobin; AFP, α-fetoprotein; ALT, alanine aminotransferase; AST, aspartate aminotransferase; TB, serum total bilirubin; ALB, albumin, GGT, gamma-glutamyl transferase; PT, prothrombin time; Scr, serum creatinine; PVTT, portal vein tumor thrombus

### Feature extraction

For R model features selection, a total of 112 radiomics features were extracted and normalized. A total of 18 features with nonzero coefficients in the LASSO regression model were selected, including two image-original related features, six shape-related features, two first-order features, and eight textural features. For the C model’s features, four features were selected: PVTT, tumor number, AFP, and GGT.

The R-C model consisted of 13 features with nonzero coefficients in LASSO regression model, including 10 radiomics features and three clinical features. The clinical features were as follows: PVTT, tumor number, and AFP. The radiomics features included one image-related feature, three shape-related features, one first-order feature, and five textural features. Moreover, 8 radiomics features comprised the R and R-C models.

The importance of selected features was showed in Figure S2. The detailed features of three models were listed in Table S2.

### Predictive Model Development and Validation

**Fig. 3:**
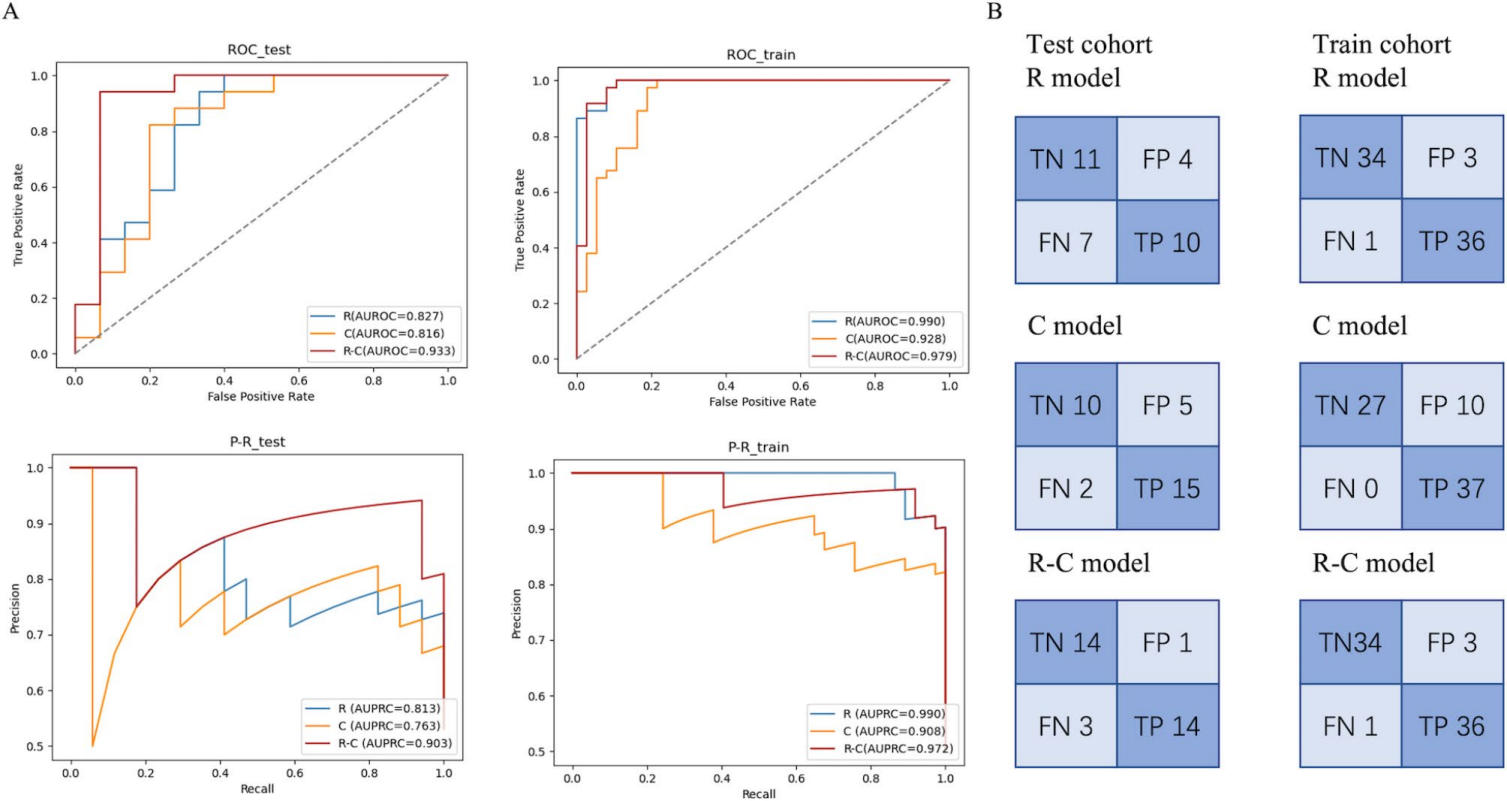
Performance of the R model, C model, and R-C model (A) ROC and P-R curves of the three models in test and training cohorts (B) Confusion matrix of the three models in the test and training cohorts

We adopted the SVM algorithm to establish three classification models for predicting HCC downstaging outcomes with the features selected above: R, C, and R-C. Next, we tested the performance of different models in the same training and test cohorts. The training cohort consisted of 74 (70%) cases, and the test cohort consisted of 32 (30%) cases. In the test cohort, the accuracy of the R model, C model, and R-C model was 0.656, 0.781, and 0.875, and the AUROC of the three models was 0.827, 0.816, and 0.933, respectively. The ROC, P-R curve, and confusion matrix of the test and training cohorts of the different models are shown in Fig. 3. Precision, recall, and F1-score are shown in Table 3. While the Delong test indicated that there was no significant difference in AUROC between each pair of models in the test cohort (Table S3). It is noteworthy that the R-C model demonstrated notably improved accuracy compared to the R model (p = 0.039, Table S4). The R model, C model, and R-C model showed good performance; however, the R-C model had better prognostic ability than the others.

**Table 3:** Performance in test and train cohorts of three models

			Precision	Recall	F1-score
**Test cohort**	R model	0	0.61	0.73	0.67
		1	0.71	0.59	0.65
	C model	0	0.83	0.67	0.74
		1	0.75	0.88	0.81
	R-C model	0	0.82	0.93	0.87
		1	0.93	0.82	0.87
**Train cohort**	R model	0	0.97	0.92	0.94
		1	0.92	0.97	0.95
	C model	0	1.00	0.73	0.84
		1	0.79	1.00	0.88
	R-C model	0	0.97	0.92	0.94
		1	0.92	0.97	0.95

0, failure;1, success

**Fig. 4:**
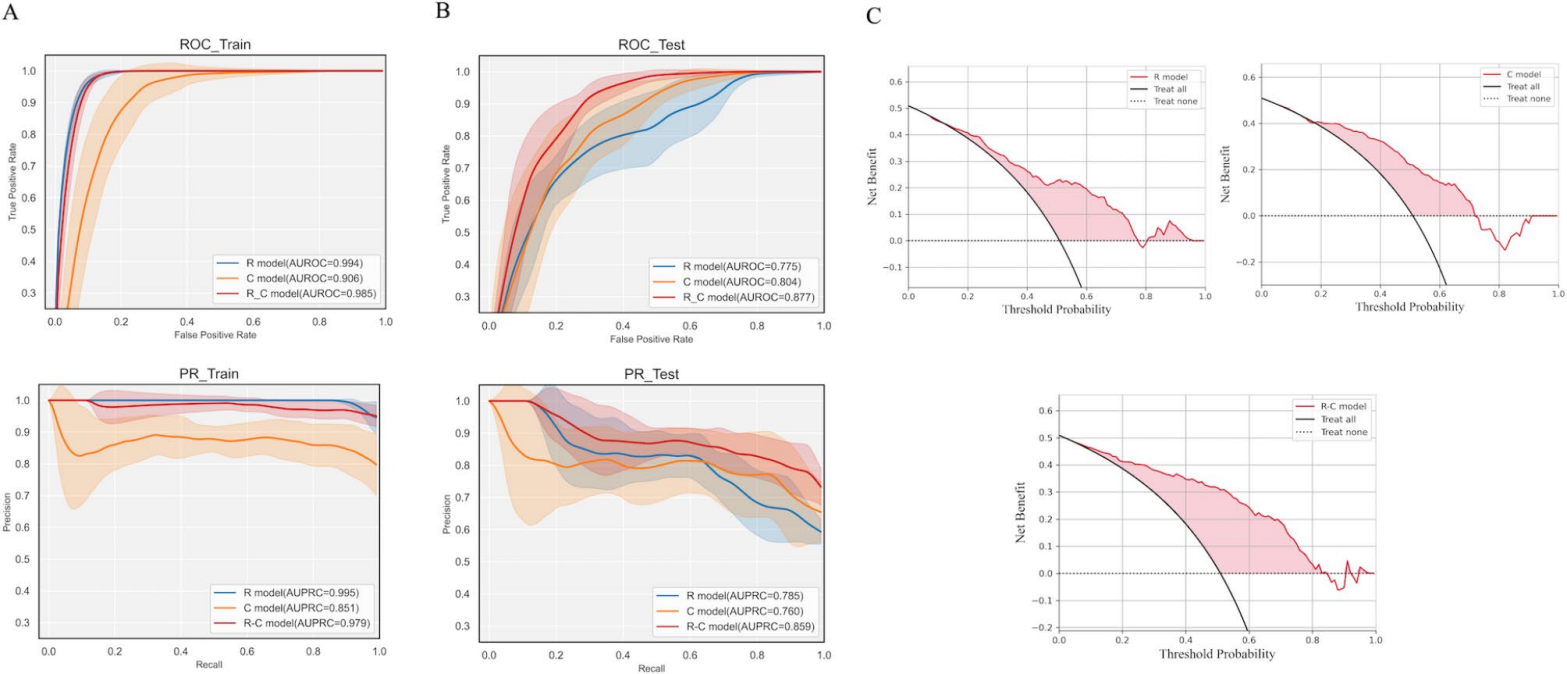
K-fold cross-validation (A) ROC of three models in the test and training cohorts (B) P-R curve of three models in the test and training cohorts (C) DCA of three models in the test cohorts

Herein, we adopted k-fold cross-validation to test the stability of the three models. After k-fold cross-validation (k = 3, repeat = 2), all three models showed stable performance in the test cohort and training cohorts (see Fig. 4A and B). In the test cohort, the average accuracy of the R, C, and R-C models was 0.712, 0.792, and 0.844, the average AUROC of the three models was 0.775, 0.804, and 0.877, and the average AUPRC of the three models was 0.785, 0.760, and 0.859, respectively. Figure 4C showed the DCA of the three models in the test cohort.

### Inspection of model features

**Fig. 5:**
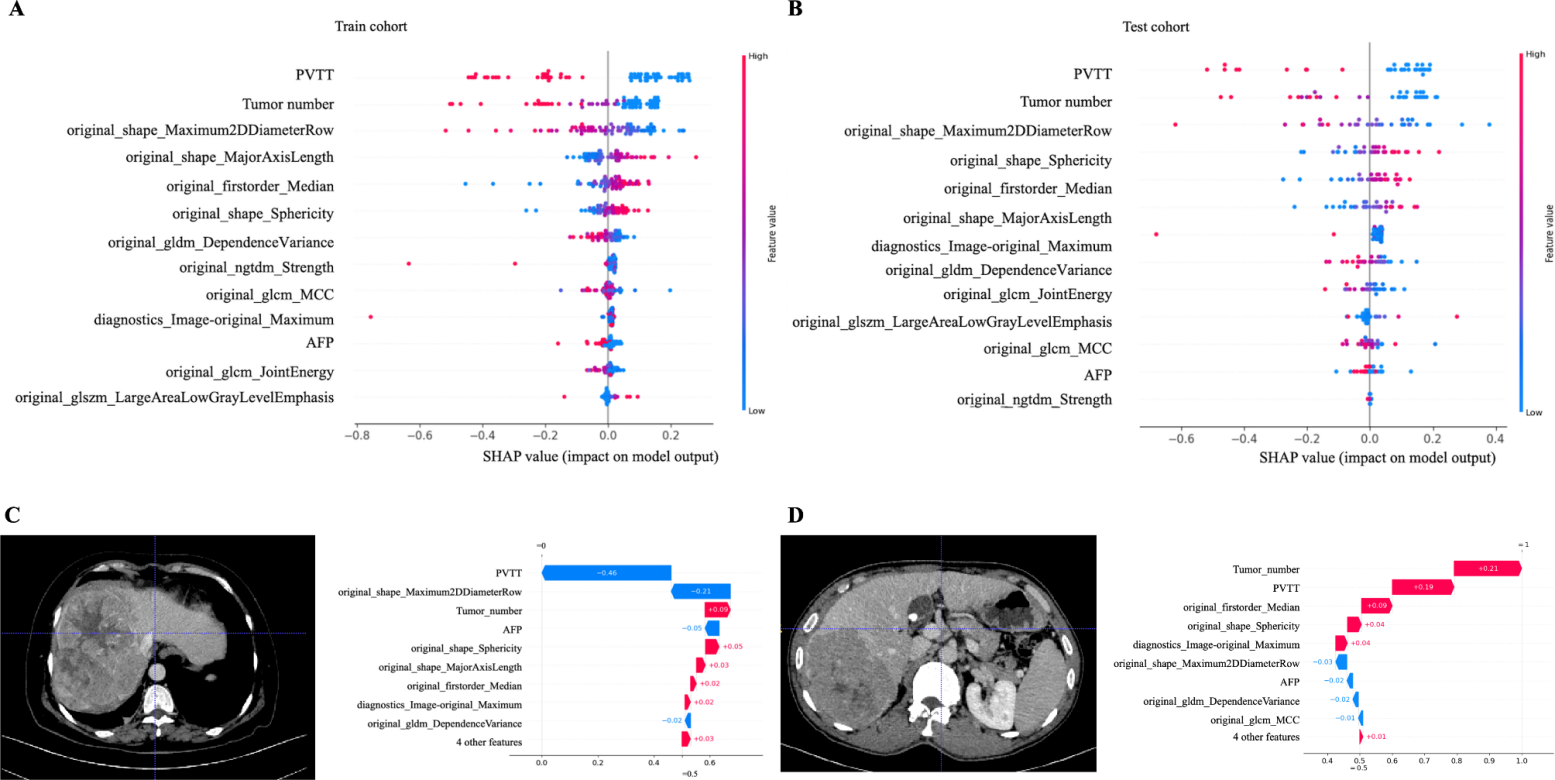
Feature inspection (A) Feature inspection in specific R-C model (Train cohort) (B) Feature inspection in specific R-C model (Test cohort) (C-D) Feature inspection in specific patient

Feature importance was also assessed using SHAP values in the trained R-C SVM model (see Fig. 5). The impact of each key feature on the model prediction was shown in SHAP values. For different trained models, the SHAP values of features may not be consistent. However, the importance of tumor burden (such as tumor diameter, tumor number, and PVTT) was stable in different cohorts. However, if we only used tumor burden to build the predictive model, accuracy and AUROC were poorer than the R-C model (Figure S1). The average accuracy and AUROC were 0.769 and 0.792, respectively, when the tumor burden parameter was used to build the model; in the same test cohort, the average accuracy and AUROC of the R-C model were 0.788 and 0.814, respectively.

## Discussion

In this study, the R-C model was proposed for the first time, which accurately predicted the downstaging outcomes of HCC patients in LT. Previous clinical studies focused on the survival after LT within successful downstaging; however, only a few studies concentrated on predicting the downstaging response in HCC patients [4, 13, 14, 22]. Since the reported downstaging failure rates varied, predicting the outcomes of pre-treatment in HCC patients provided individual treatment strategies.

The biological nature of a tumor involves multiple interacting components, which might be reflected when considering various features [23]. In the current study, we constructed the R model, C model, and R-C model with k-fold cross-validation. The average accuracy of the three models was 0.712, 0.792, and 0.844, the average AUROC of the three models was 0.775, 0.804 and 0.877, and the average AUPRC of the three models was 0.785, 0.760 and 0.859, respectively. In terms of incorporating wavelet transform features, we performed a comprehensive comparative analysis of R model and original radiomics features with wavelet transform features model (R_w model). We filtered to obtain 18 and 22 features from R features and R_w features, respectively (Table S5). Upon meticulous evaluation of the results presented in Table S6 and Figure S3, it was evident that the R_w model did not surpass the performance of the R model on the ROC (p = 0.2291) and P-R curves. To strike a balance between interpretability and effectiveness in the model, the focus of this study was on choosing the most representative R model features instead of R_w ones. The R-C model, with better accuracy, AUROC and AUPRC, showed more stability than the other models. The R-C model consisted of clinical data, and radiomics features performed better than previous studies. Based on objective multidimensional parameters, the R-C model is considered an effective, accurate, and intelligent method that accurately predicts the downstaging success of HCC patients in LT.

Nowadays, radiomics revealed tumor heterogeneities, which made it possible to study the correlation between radiomics features and downstaging outcomes [20, 24–26]. In our R-C model, we identified 10 predictors from 112 radiomics features. Four features were tumor morphological-related, including diameter, axis length (least and major), and sphericity. In addition to tumor size-related features, we also found that shape_sphericity was positively correlated to the downstaging outcomes, and the regular shape indicated good tumor biological behavior. Besides these, the other eight features were image original features, first order features and textural features.

Some radiomics features, which had predictive implications that were not reported before, implied tumor heterogeneity and were difficult to identify by radiologists and physicians [20, 27]. To the best of our knowledge, although studies speculated that morphological features might be predictors of HCC downstaging outcomes, no study has demonstrated the correlation between radiomics and downstaging outcomes. However, the performance of the R model was not as stable as the R-C model, which might be because radiomics features could reveal tumor heterogeneities but failed to reflect liver function and macrovascular invasion, which is essential for outcomes [28, 29]. Thus, in the R-C model, we used clinical features to improve the accuracy of the prediction model.

In a review of downstaging for LT, most studies showed that Child–Pugh class and tumor burden were associated with the outcome of downstaging [2, 14, 16, 30–33]. Such studies focused on the differences between successful and failed downstaging groups but lacked a test cohort. One liver function-related feature (GGT) in our C model was considered a predictive feature. Based on the current study, the Child–Pugh class was not the predictive feature, which might be because the laboratory parameters were more objective in assessing liver function compared to Child–Pugh class [2, 31, 32]. Previous studies demonstrated that when used alone, AFP has low sensitivity in HCC surveillance; however, imaging in combination with AFP reached optimal sensitivity [34]. In the current study, the AFP was critical in different outcomes of downstaging, and AFP was one of the predictors in the R-C model. Previous studies recommended that tumor burden (such as tumor size, tumor number, tumor volume, or macrovascular invasion) was related to HCC survival, while some studies used tumor burden as a predictor of survival in patients who underwent transcatheter arterial chemoembolization [14, 30–33, 35]. However, the tumor size was controversial, and some studies found that necrosis was high in large tumors [36, 37]. In our study, the tumor burden-related features were critical in the R-C model, especially tumor diameter, tumor number, and PVTT. This finding was in agreement with previous studies. However, when we constructed a predictive model, only tumor diameter, tumor number, and PVTT were used, the predictive ability was limited. This indicated that the radiomics features were necessary for enhanced performance.

Nevertheless, the present study had some limitations. Firstly, it was a small patient cohort encompassing two centers, and the predictability of the tumor downstaging treatment was beyond the scope of our study. Secondly, it was a retrospective study but exhibited a potential predicted value. Further, a multicenter clinical trial should be designed to examine the R-C model and focus on finding the standardized downstaging protocol.

In conclusion, we investigated the downstaging outcomes of HCC patients for LT by analyzing the clinical data and radiomics features. The novel and practical R-C model accurately predicted the downstaging outcomes and could be applied as guidance for the downstaging treatment in the future.

### Electronic supplementary material

Below is the link to the electronic supplementary material.

Supplementary Material 1

Supplementary Material 2

Supplementary Material 3

Supplementary Material 4

Supplementary Material 5

## Data Availability

The data are available from the corresponding author on reasonable request.

## Abbreviations

HCC: Hepatocellular carcinoma
LT: Liver transplantation
3D: Three-dimensional
OS: Overall survival
RFS: Recurrence-free survival
CT: Computed tomography
BMI: Body mass index
SVM: Support vector machine
R: Radiomics features
C: Clinical features
R-C: Radiomics-clinical features
MVI: Microvascular invasion
ML: Machine learning
mRECIST: modified Response Evaluation Criteria in Solid Tumors
WBCs: White blood cells
PLT: Platelet count
Hb: Hemoglobin
AFP: Alpha-fetoprotein
ALT: Alanine aminotransferase
AST: Aspartate aminotransferase
TB: Serum total bilirubin
ALB: Albumin
GGT: Gamma-glutamyl transferase
PT: Prothrombin time
Scr: Serum creatinine
ROI: Regions of interest
LASSO: Least absolute shrinkage and selection operator
ROC: Receiver-operating characteristic
AUROC: Area under the ROC curve
P-R curve: Precision-recall curve
AUPRC: Area under the P-R curve
SHAP: SHapley Additive exPlanations
TP: True negative
TN: True negative
FN: False negative
FP: False negative
DCA: Decision curve analysis
PVTT: Portal vein tumor thrombus
