# Supplementary figures and images for: Predicting the outcomes of hepatocellular carcinoma downstaging with the use of clinical and radiomics features
Source: BMC Cancer. 2023 Sep 12;23:858. doi: 10.1186/s12885-023-11386-0 (PMC10496191; doi:10.1186/s12885-023-11386-0)

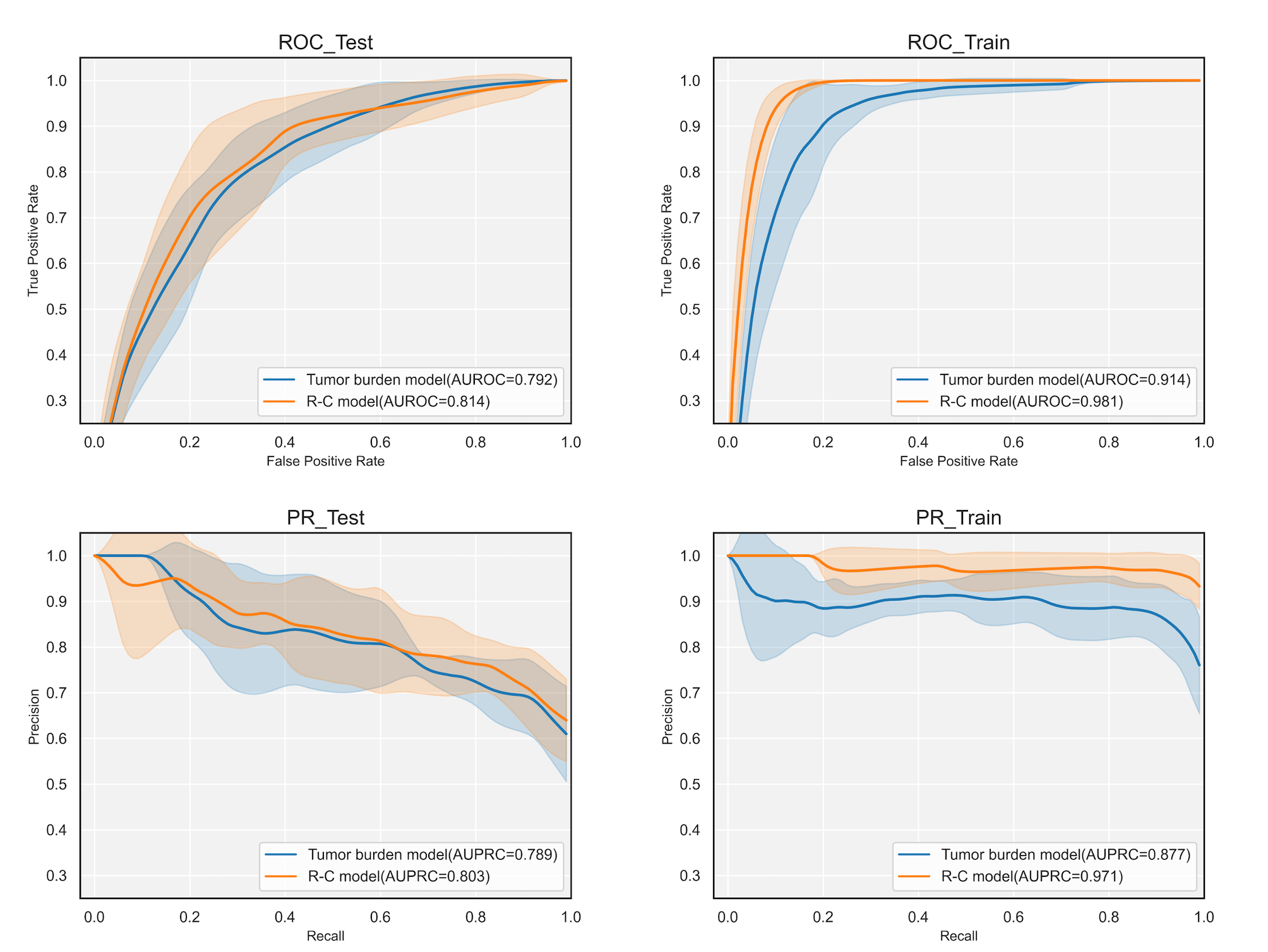

Supplement: Supplementary file 1 — Supplementary Material 1 [file 12885_2023_11386_MOESM1_ESM.png]

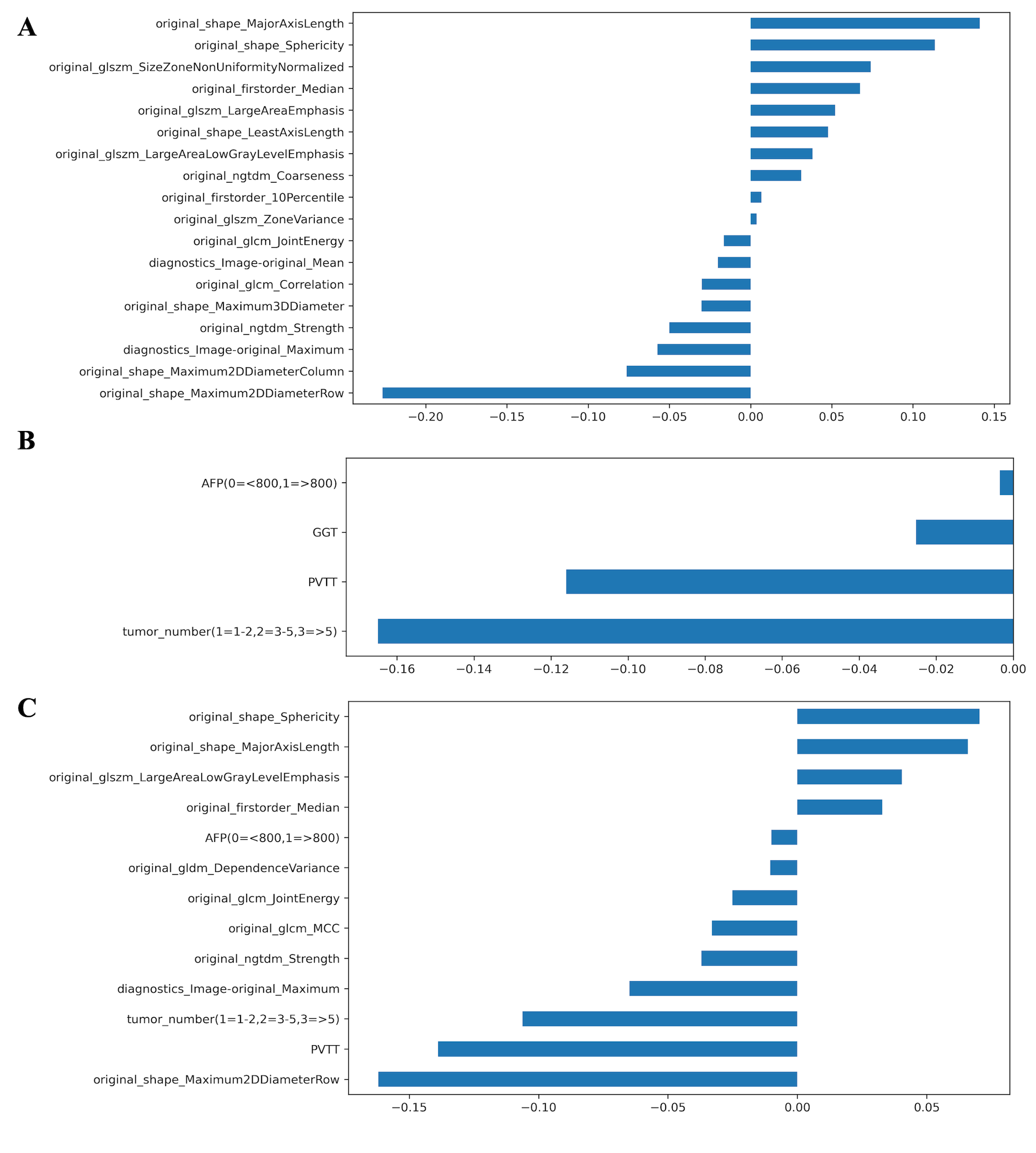

Supplement: Supplementary file 2 — Supplementary Material 2 [file 12885_2023_11386_MOESM2_ESM.png]

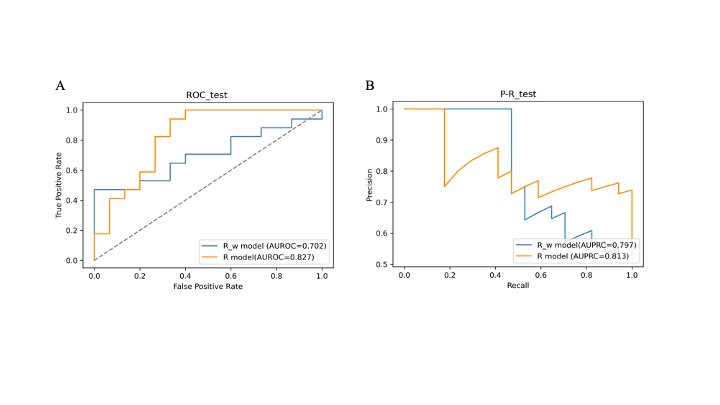

Supplement: Supplementary file 3 — Supplementary Material 3 [file 12885_2023_11386_MOESM3_ESM.png]
