# Supplementary material for: Predicting the outcomes of hepatocellular carcinoma downstaging with the use of clinical and radiomics features
Source: BMC Cancer. 2023 Sep 12;23:858. doi: 10.1186/s12885-023-11386-0 (PMC10496191; doi:10.1186/s12885-023-11386-0)
Supplement: Supplementary file 4 — Supplementary Material 4 [file 12885_2023_11386_MOESM4_ESM.docx]

Supplementary figure legends

**Figure S1.** K-fold cross-validation of tumor burden model and R-C model

**Figure S2.** Features coefficients in the LASSO regression model

(A) R model features coefficients

(B) C model features coefficients

(C) R-C model features coefficients

**Figure S3.** Performance of the R_w model and R model.

(A) ROC curves of the two models in test cohorts.

(B) P-R curves of the two models in test cohorts.
