# Supplementary material for: Predicting the outcomes of hepatocellular carcinoma downstaging with the use of clinical and radiomics features
Source: BMC Cancer. 2023 Sep 12;23:858. doi: 10.1186/s12885-023-11386-0 (PMC10496191; doi:10.1186/s12885-023-11386-0)
Supplement: Supplementary file 5 — Supplementary Material 5 [file 12885_2023_11386_MOESM5_ESM.docx]

Supplementary tables

Table S1 Downstaging Treatments

| Therapy | N=106 |
| --- | --- |
| Local therapy only |  |
| TACE/HAIC | 74 |
| TACE+HAIC | 8 |
| SBRT | 3 |
| TACE/HAIC+SBRT | 3 |
| Local therapy +Systemic therapy |  |
| Local therapy + TKI | 11 |
| Local therapy + PD-1 | 1 |
| Local therapy + TKI + PD-1 | 6 |

TACE : transarterial chemoembolization

HAIC : hepatic arterial infusion chemotherapy

SBRT : stereotactic body radiation therapy

TKI : tyrosine kinase inhibitors

PD-1 : programmed cell death 1

Table S2 Features of the three model

| Features | R model | C model | R-C model |
| --- | --- | --- | --- |
| PVTT |  | √ | √ |
| Tumor number |  | √ | √ |
| AFP |  | √ | √ |
| GGT |  | √ |  |
| Image-original_Mean | √ |  |  |
| Image-original_Maximum | √ |  | √ |
| original_shape_LeastAxisLength* | √ |  |  |
| original_shape_MajorAxisLength* | √ |  | √ |
| original_shape_Maximum2DDiameterRow* | √ |  | √ |
| original_shape_Sphericity | √ |  | √ |
| original_firstorder_10Percentile | √ |  |  |
| original_firstorder_Median | √ |  | √ |
| original_glcm_MCC |  |  | √ |
| original_gldm_DependenceVariance |  |  | √ |
| original_glszm_LargeAreaEmphasis | √ |  |  |
| original_glszm_LargeAreaLowGrayLevelEmphasis | √ |  | √ |
| original_ngtdm_Strength | √ |  | √ |
| original_shape_Maximum2DDiameterColumn* | √ |  |  |
| original_shape_Maximum3DDiameter* | √ |  |  |
| original_glcm_Correlation | √ |  |  |
| original_glcm_JointEnergy | √ |  | √ |
| original_glszm_SizeZoneNonUniformityNormalized | √ |  |  |
| original_ngtdm_Coarseness | √ |  |  |
| original_glszm_ZoneVariance | √ |  |  |

*unit: voxel as unit

PVTT Portal Vein Tumor Thrombus

AFP Alpha Fetoprotein

GGT Gamma-glutamyl transferase

Table S3. Delong test of the three models in test cohort

| Model | Z | p |
| --- | --- | --- |
| R model vs C model | 0.10085 | 0.9197 |
| R-C model vs C model | 1.5261 | 0.1270 |
| R-C model vs R model | 1.4813 | 0.1385 |

Table S4. Statistical comparison of accuracy of the three models in test cohort

|  |  | Correct Cases | Incorrect Cases | p value |
| --- | --- | --- | --- | --- |
| R model vs C model | R model | 21(65.6%) | 11(34.4%) | 0.266 |
|  | C model | 25(78.1%) | 7(21.9%) |  |
| R-C model vs C model | R-C model | 28(87.5%) | 4(12.5%) | 0.320 |
|  | C model | 25(78.1%) | 7(21.9%) |  |
| R-C model vs R model | R-C model | 28(87.5%) | 4(12.5%) | 0.039 |
|  | R model | 21(65.6%) | 11(34.4%) |  |

Table S5 Features of the R model with/without wavelet filters

| Features | R model | R_w model |
| --- | --- | --- |
| Image-original_Mean | √ |  |
| Image-original_Maximum | √ |  |
| original_shape_LeastAxisLength* | √ |  |
| original_shape_MajorAxisLength* | √ |  |
| original_shape_Maximum2DDiameterRow* | √ | √ |
| original_shape_Sphericity | √ | √ |
| original_firstorder_10Percentile | √ |  |
| original_firstorder_Median | √ |  |
| original_glszm_LargeAreaEmphasis | √ |  |
| original_glszm_LargeAreaLowGrayLevelEmphasis | √ |  |
| original_ngtdm_Strength | √ |  |
| original_shape_Maximum2DDiameterColumn* | √ |  |
| original_shape_Maximum3DDiameter* | √ |  |
| original_glcm_Correlation | √ |  |
| original_glcm_JointEnergy | √ |  |
| original_glszm_SizeZoneNonUniformityNormalized | √ |  |
| original_ngtdm_Coarseness | √ |  |
| original_glszm_ZoneVariance | √ |  |
| LLH_original_shape_Maximum2DDiameterRow |  | √ |
| LLH_original_shape_Sphericity |  | √ |
| LLH_original_glcm_Idm |  | √ |
| LLH_original_gldm_SmallDependLowGrayLevelEmphasis |  | √ |
| LHL_original_shape_Maximum2DDiameterRow |  | √ |
| LHL_original_shape_Sphericity |  | √ |
| LHH_original_shape_Sphericity |  | √ |
| LHH_original_glcm_Imc1 |  | √ |
| HLL_original_shape_Sphericity |  | √ |
| HLH_original_firstorder_Kurtosis |  | √ |
| HLH_original_glcm_Id |  | √ |
| HLH_original_glcm_InverseVariance |  | √ |
| HHL_original_shape_Maximum2DDiameterRow |  | √ |
| HHL_original_shape_Sphericity |  | √ |
| HHL_original_glrlm_LongRunHighGrayLevelEmphasis |  | √ |
| HHH_original_shape_Maximum2DDiameterRow |  | √ |
| HHH_original_shape_Sphericity |  | √ |
| HHH_original_glszm_SizeZoneNonUniformityNormalized |  | √ |
| HHH_original_glszm_ZoneEntropy |  | √ |
| LLL_original_firstorder_Median |  | √ |

*unit: voxel as unit

R model : without wavelet filters

R_w model : with wavelet filters

Table S6 Performance of the R model with/without wavelet filters (test cohort)

|  | R model | R_w model | p |
| --- | --- | --- | --- |
| Accuracy | 0.656(21/32) | 0.625(20/32) | 0.794 |
| AUROC | 0.827 | 0.702 | 0.2291 |

R model : without wavelet filters

R_w model : with wavelet filters
